# Gene-level differential analysis at transcript-level resolution

**DOI:** 10.1186/s13059-018-1419-z

**Published:** 2018-04-12

**Authors:** Lynn Yi, Harold Pimentel, Nicolas L. Bray, Lior Pachter

**Affiliations:** 1 UCLA-Caltech Medical Science Training Program, Los Angeles, CA USA; 2 Division of Biology and Biological Engineering, Caltech, Pasadena, CA USA; 3 Department of Genetics, Stanford University, Palo Alto, CA USA; 4 Innovative Genomics Institute, Berkeley, CA USA; 5 Department of Computing and Mathematical Sciences, Caltech, Pasadena, CA USA

**Keywords:** RNA-sequencing, Differential expression, Meta-analysis, *P* value aggregation, Lancaster method, Fisher’s method, Šidák correction, RNA-seq quantification, RNA-seq alignment, Pseudoalignment, Transcript compatibility counts, Gene ontology

## Abstract

Compared to RNA-sequencing transcript differential analysis, gene-level differential expression analysis is more robust and experimentally actionable. However, the use of gene counts for statistical analysis can mask transcript-level dynamics. We demonstrate that ‘*analysis first, aggregation second,*’ where the *p* values derived from transcript analysis are aggregated to obtain gene-level results, increase sensitivity and accuracy. The method we propose can also be applied to transcript compatibility counts obtained from pseudoalignment of reads, which circumvents the need for quantification and is fast, accurate, and model-free. The method generalizes to various levels of biology and we showcase an application to gene ontologies.

**Electronic supplementary material:**

The online version of this article (10.1186/s13059-018-1419-z) contains supplementary material, which is available to authorized users.

## Background

Direct analysis of RNA abundance by sequencing complementary DNAs (cDNAs) using RNA-sequencing (RNA-seq) offers the possibility of analyzing expression at the resolution of individual transcripts [1]. Nevertheless, RNA-seq continues to be mostly studied at the gene level, partly because such analyses appear to be more robust [2] and also because gene-level discoveries are more experimentally actionable than transcript-level discoveries due to the difficulty of knocking down single isoforms [3].

Gene-level RNA-seq differential analysis is, at first glance, similar to transcript-level analysis, with the caveat that transcript counts are first summed to obtain gene counts [4, 5]. However, despite such superficial simplicity, there is considerable complexity involved in transitioning from transcripts to genes. In [6], it was shown that a naïve approach of summing transcript counts to gene counts leads to inaccurate estimates of fold-change between conditions when transcripts have different lengths. Because transcript counts are proportional to transcript lengths, summing transcript counts is not equivalent to summing transcript abundances.

**Fig. 1:**
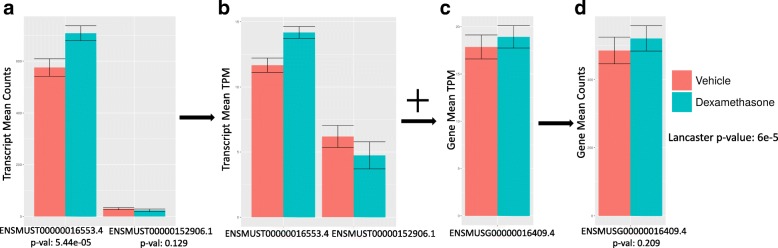
Conversion of transcript counts to gene counts for the *Nkap* gene in the dexamethasone dataset under two conditions (dexamethasone and vehicle treatment). The *x*-axis is labeled with the Ensembl gene and transcript IDs, along with *p* values obtained by performing sleuth on transcripts and genes. In this process, the transcript counts (**a**) are converted into transcript abundances (**b**) by normalization according to transcript lengths. Transcript abundances are then summed to obtain gene abundances (**c**) and then converted to gene counts (**d**) using the median or mean transcript length as a proxy for the gene length. The converted gene counts mask significant changes among the constituent transcripts and the gene count variance does not directly reflect the combined variance in transcript counts. In this example, *Nkap* is not differential when examined using the converted gene counts, but can be identified as differential when the *p* values of the constituent transcripts are aggregated using the Lancaster method

**Fig. 2:**
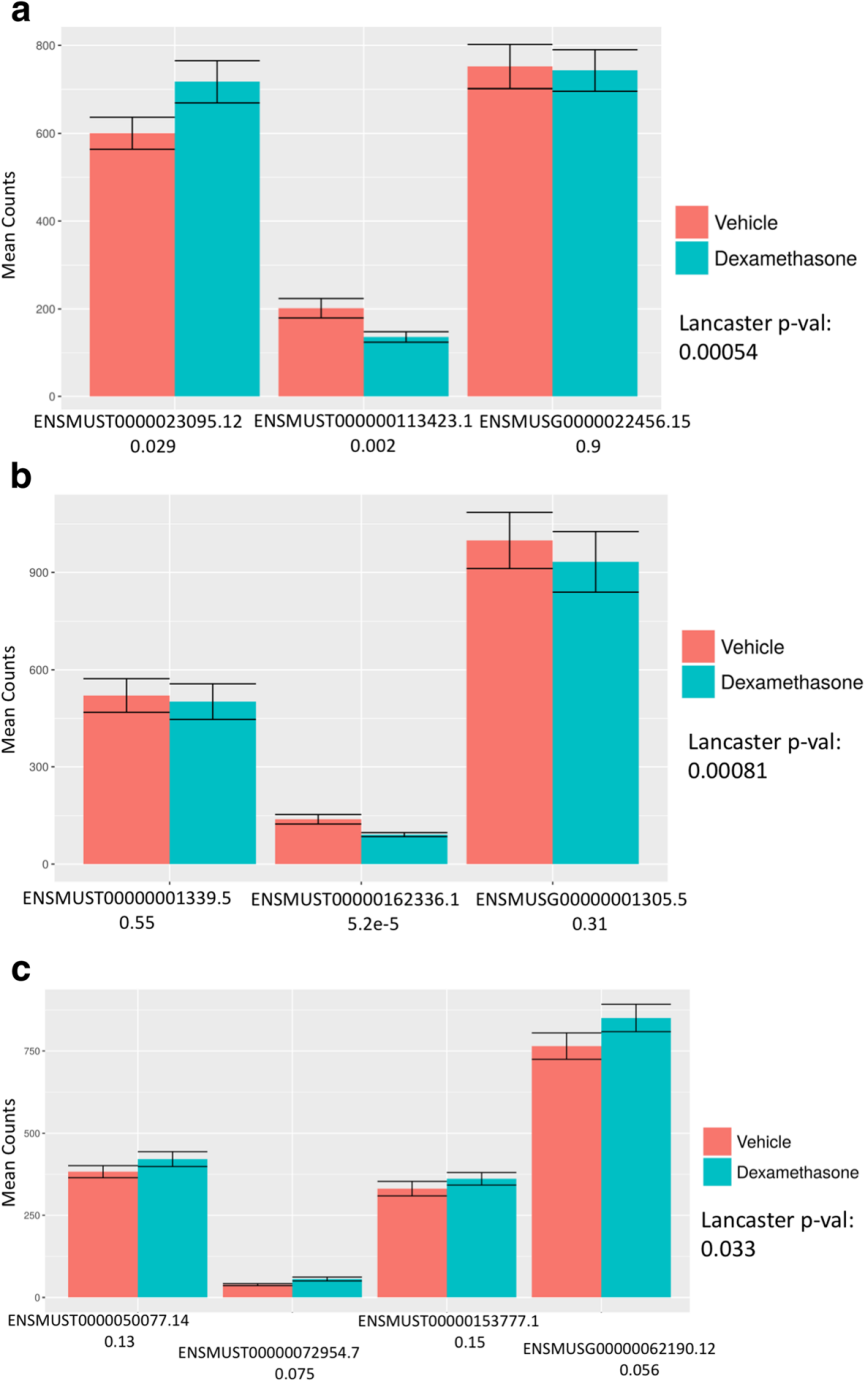
Differential transcript masking. Dynamics among transcripts may not be detected with gene-level analyses due to cancellation (**a**), domination (**b**), and collapsing (**c**). Gene counts and constituent transcript counts are plotted between conditions (dexamethasone vs vehicle treatment) and annotated with Ensembl ID and sleuth-derived *p* values. In the case of cancellation (**a**), the abundance of transcripts changing in opposite directions cancels out upon conversion to gene abundance. In domination (**b**), an abundant transcript that is not changing can mask substantial change in abundance of a minor transcript. In the case of collapsing (**c**), multiple isoforms of a gene with small effect sizes in the same direction do not lead to a significant change when observed after summation, but their independent changes constitute substantial evidence for differential expression. In all these examples, gene-level differential analysis with sleuth failed to identify the genes as differential (*p* values listed on *x*-axis), whereas Lancaster aggregation of transcript *p* values resulted in detection of the genes as differential

A remedy to this problem is to estimate gene abundances (e.g. in transcript-per-million units) by summing transcript abundances [7], but regularization methods for variance estimation of gene counts [8] cannot be directly applied to abundances. For this reason, recent workflows for gene-level differential analysis rely on converting gene abundance estimates to gene counts [2, 9]. Such methods have two major drawbacks. First, even though the resulting gene counts can be used to accurately estimate fold changes, the associated variance estimates can be distorted (see Fig. 1 and Additional file 1: Section 1). Second, the assignment of a single numerical value to a gene can mask dynamic effects among its multiple constituent transcripts (Fig. 2). In the case of “cancellation” (Fig. 2a), the abundance of transcripts changing in opposite directions cancels out upon conversion to gene abundance. In “domination” (Fig. 2b), an abundant transcript that is not changing can mask substantial change in abundance of a minor transcript. Finally, in the case of “collapsing” (Fig. 2c), due to over-dispersion in variance, multiple isoforms of a gene with small effect sizes in the same direction do not lead to a significant change when observed in aggregate, but their independent changes constitute substantial evidence for differential expression. As shown in Fig. 2, these scenarios are not only hypothetical scenarios in a thought experiment, but events that occur in biological data.

Rather than aggregating quantifications before differential analysis, one approach is to first perform a transcript-level differential analysis followed by a gene-level meta-analysis. Such a method is implemented in the DEXSeq program [10], although it is not effective at recovering differential events lost due to collapsing and is suboptimal even for cancellation or domination events (see “Results” and Additional file 1: Section 2). Meta-analysis has been suggested for microarray studies to aggregate probe-level *P* values [11] and is performed in genome-wide association studies to aggregate single nucleotide polymorphism *P* values to make gene-level [12–14] and pathway-level inferences [14, 15], but such approaches do not appear to have been extensively explored for RNA-seq.

We present a new framework for gene-level differential analysis that utilizes the Lancaster method [16]. In this framework, differential expression is performed on transcripts as usual, but then transcript-level *p* values are aggregated to obtain gene-level *p* values. (See “Methods” for details about the Lancaster method. See Additional file 1 for applicability of the Lancaster method to RNA-seq.)

Our approach can be based on *p* values derived from transcript-level differential analysis, but can also be applied to *p* values derived from comparisons of transcript compatibility counts (TCCs), a concept introduced by the pseudoalignment method in kallisto [17]. TCCs are the number of reads that are compatible with a set of transcripts, i.e. an equivalence class. In default RNA-seq quantification mode, kallisto matches each read with its equivalence class, thus generating TCCs, and then applies the expectation-maximization (EM) algorithm on TCCs to obtain transcript quantifications. Differential analysis performed on directly TCCs has the advantage of being fast and model-free, and we show that it is particularly useful for positionally biased RNA-seq data.

Finally, we highlight the generality of our approach at varying levels of biological resolution by extending it to gene ontology analysis. In contrast to classical gene ontology (GO) tests that identify enrichment of GO terms with respect to gene lists, our approach identifies GO terms in which there is significant perturbation among the associated genes. We combine this idea with TCC-based differential analysis to illustrate how GO analysis can be performed on RNA-seq data without transcript quantification.

## Results

We first examined the performance of aggregation in comparison to standard gene-level differential expression methods using three simulated scenarios from Pimentel et al. [9]. In these simulations, transcripts are perturbed independently, in a correlated fashion with other transcripts of the gene, or according to effect sizes observed in a biological experiment. In the first scenario of independent effects, random transcripts in the transcriptome are independently chosen to be perturbed and the effect size for each transcript is chosen independently. In the second scenario of correlated effects, genes are independently chosen to be differentially expressed and all transcripts of the same gene are perturbed in the same direction. In the third scenario of experimentally based effects, effect sizes are learned from an experimental dataset and applied to the simulation (see “Methods” for more details). Each of the three scenarios was simulated 20 times.

We evaluated the performance of various aggregation methods on these simulations with two differential expression methods: sleuth and DESeq2. These differential expression methods were chosen for their superior performance in previously published simulations [9]*.* sleuth utilizes bootstraps on reads to estimate inferential variance due to read-mapping and quantification uncertainty, which is then used in a linear model to perform differential expression analysis. DESeq2 utilizes a negative binomial model on counts [18]. We evaluated every aggregation method using each differential expression method in each of the three simulation scenarios.

**Fig. 3:**
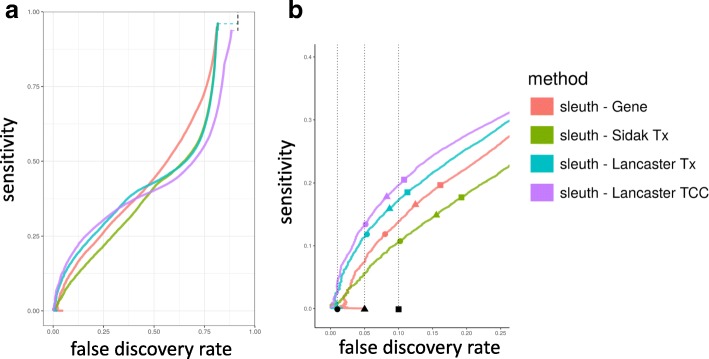
Sensitivity and false discovery trade-off curves of aggregation methods. Twenty simulated experiments based on parameters estimated from biological data were analyzed with different aggregation methods and averaged producing (**a**) and zoomed in (**b**). sleuth in gene mode (“sleuth-Gene”) is a standard gene-level differential analysis method. Aggregation results based on transcript *p* values are shown using two approaches: sleuth transcript *p* values aggregated by the Lancaster method (“sleuth-Lancaster Tx”) and sleuth transcript *p* values aggregated by the Šidák-adjusted minimum method (“sleuth – Sidak Tx”). Finally, sleuth TCC *p* values obtained by running sleuth on TCC counts were aggregated with the Lancaster method (“sleuth-Lancaster TCC”). *Dashed lines* indicate true FDR at 0.01, 0.05, and 0.1. The shapes (*circle*, *triangle*, *square*) on each sensitivity-FDR curve indicate the true FDR and sensitivity at each method’s reported FDRs of 0.01, 0.05, and 0.1

Figure 3 shows the results of performing aggregation using sleuth in the simulation scenario that is modeled after experimental effect sizes, plotted as a false discovery rate (FDR)-sensitivity tradeoff curve. (Additional File 1: Figures S1 and S2 show results with other two simulation scenarios using sleuth. Additional File 1: Figure S3 shows results with the three simulation scenarios using DESeq2.) Aggregation of transcript *p* values using the Lancaster method [16] outperforms standard gene-level analysis; it provides greater power at lower FDR. Furthermore, Lancaster-based aggregation outperforms the Šidák method of DEXSeq, which utilizes the minimum transcript *p* value to make the gene-level determination (method corrected, Additional file 1: Section 2). While the Šidák method performs well when transcripts are perturbed independently (Additional file 1: Figure S1), it performs very poorly in the more common case of correlated effect (Additional file 1: Figure S2). In addition to providing more power at lower FDR than the other methods, the Lancaster method is also better at controlling and accurately reporting FDR (See Fig. 3b for reported FDRs). Additional file 1: Figure S3 shows similar improvements when aggregation is performed using *p* values that are derived from DESeq2 [18] instead of sleuth. Regardless of the differential expression method used to compute *p* values, the Lancaster method of aggregation outperforms the other methods, showing that improvements in performance are due to the aggregation method and not the differential expression software.

Transcript-level *p* values are computed from transcript quantifications, a process that introduces uncertainty from multiple-mapping RNA-seq reads. Pimentel et al. [9] showed that propagating uncertainty from the transcript quantification to differential expression analysis increases accuracy of the differential expression analysis. In kallisto [17], pseudoalignment was performed to generate TCCs, which are the number of reads that are compatible with sets of transcripts and therefore do not contain any quantification uncertainty. Given the improved results observed with performing Lancaster aggregation, we asked whether it is possible to perform differential expression analysis directly on TCCs and aggregate on TCC *p* values to obtain gene *p* values, thereby bypassing transcript quantification and the uncertainty it entails altogether. Figure 3 shows that aggregating TCC *p* values outperforms other methods, including that of aggregating transcript *p* values. Furthermore, aggregating TCC *p* values reported FDRs that are as or more accurate than those reported by other methods. In this instance, we used only TCCs that mapped solely to the transcripts of a single gene, which accounts for 88% of the RNA-seq reads. It may be possible to continue to improve performance by accounting for intergenic TCCs.

**Fig. 4:**
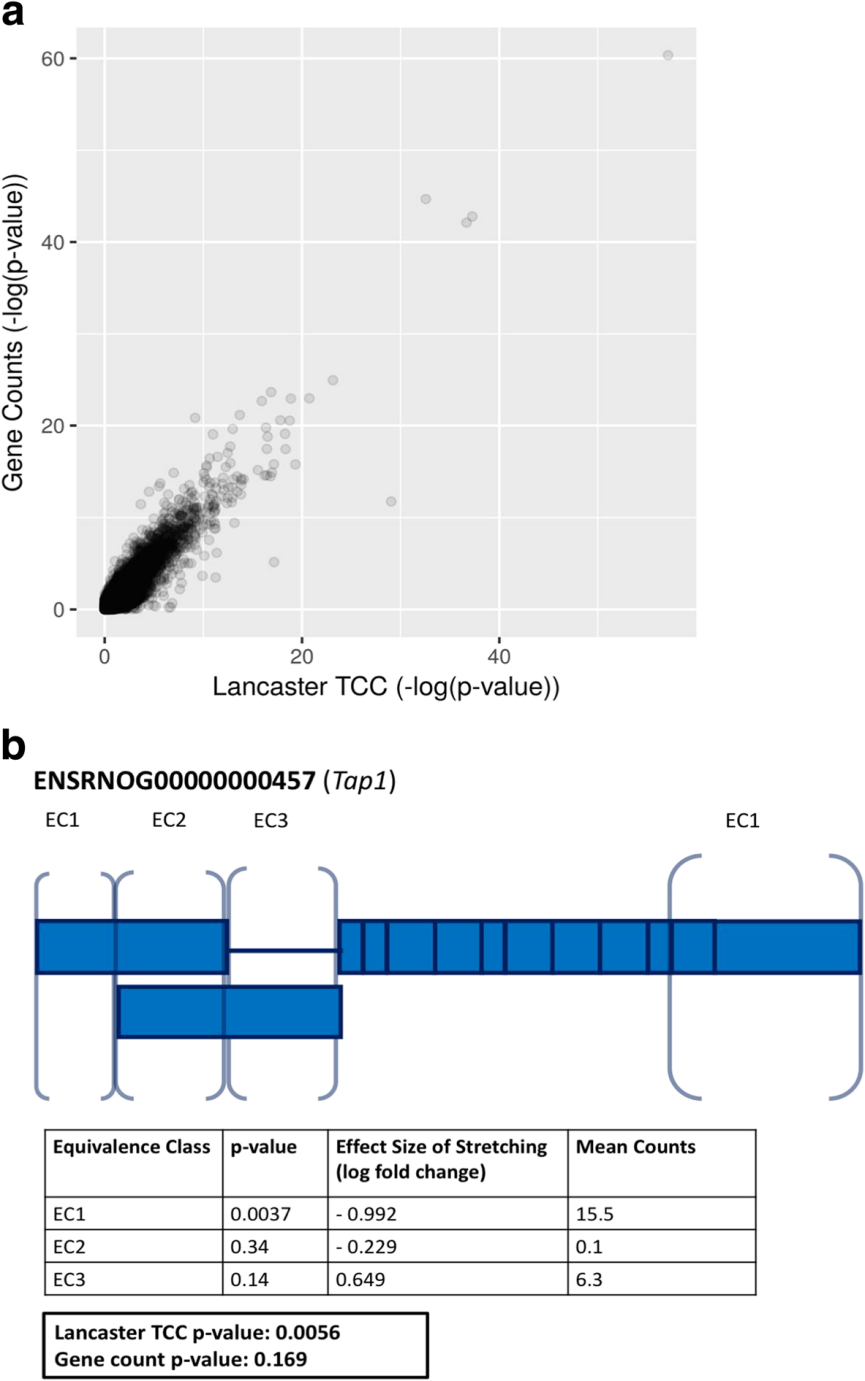
Analysis of positionally biased RNA-seq data using TCC aggregation. A log-log plot of *p* values comparing aggregated sleuth-derived TCC *p* values using the Lancaster method (*x*-axis) to *p* values obtained by differential analysis in DESeq2 with gene counts (*y*-axis) shows overall agreement (**a**). DESeq2 applied on gene counts discovered 460 DE genes (FDR < 0.05); Lancaster aggregation on TCCs discovered 243 genes (FDR < 0.05). TCC aggregated analysis can detect differential 3’ UTR usage that is masked in gene count analyses (**b**). An example is shown from the rat gene *Tap1*, with *rectangular blocks* representing individual exons (blank = non-coding, solid = coding), and distinct equivalence classes (ECs) labeled with *brackets*. Two other transcripts and their corresponding (zero count) equivalence classes are not shown. Significance levels for *Tap1* under effects of alveolar stretching were calculated using the Lancaster method (*p* value = 0.0056) and compared to *p* values derived from gene counts (*p* value = 0.169)

Aggregation of TCCs is useful when quantification is complicated due to non-uniformity of reads coverage across transcript spans. While non-uniformity in coverage is prevalent in RNA-seq [19], it is particularly extreme in variants of RNA-seq that enrich for 5′ or 3′ sequences. We used TCC aggregation to perform differential expression on QuantSeq data [20], where an experiment involved mechanically stretching rat primary type I like alveolar epithelial cells and then performing QuantSeq 3’ messenger RNA (mRNA) sequencing to detect changes in 3′ untranslated region (UTR) expression ([21], GEO Series GSE89024). Figure 4a shows that overall results with TCC-based aggregation are similar to standard analysis based on gene counts obtained by summing the number of reads that map to any constituent isoforms. However, TCC-based aggregation allows for the discovery of events that are masked in standard count-based analysis. Figure 4b shows an example where we discovered 3’ UTR isoform switching, an event which could not be identified with a gene counts-based analysis. While *p* value aggregation works well for gene-level differential expression analysis, aggregation can be extended to other natural groupings. To demonstrate the generality of the approach, we applied *p* value aggregation to gene ontologies [22]. Classic gene ontology (GO) analysis of a RNA-seq experiment involves first performing gene differential expression analysis to obtain either a list of statistically differential genes (i.e. all genes with q-value < 0.05) or a rank order list of genes (i.e. ordered by *p* value) and then identifying GOs that are statistically enriched in this gene list. Common statistical tests for enrichment include Fisher’s exact test and Wilcoxon rank-sum test [23, 24]. Instead of testing for enrichment of GOs, we examined the complementary question of “perturbation analysis,” namely, whether the GO is significantly perturbed. To test for perturbation, we aggregated *p* values based on transcript quantifications or TCCs for all genes in each GO term to obtain *p* values for each GO term, which are then Bonferroni corrected. Unlike standard GO enrichment analysis, this perturbation analysis utilizes the information derived from all genes and reveals information not only about membership, but also about the significance of perturbation.

**Fig. 5:**
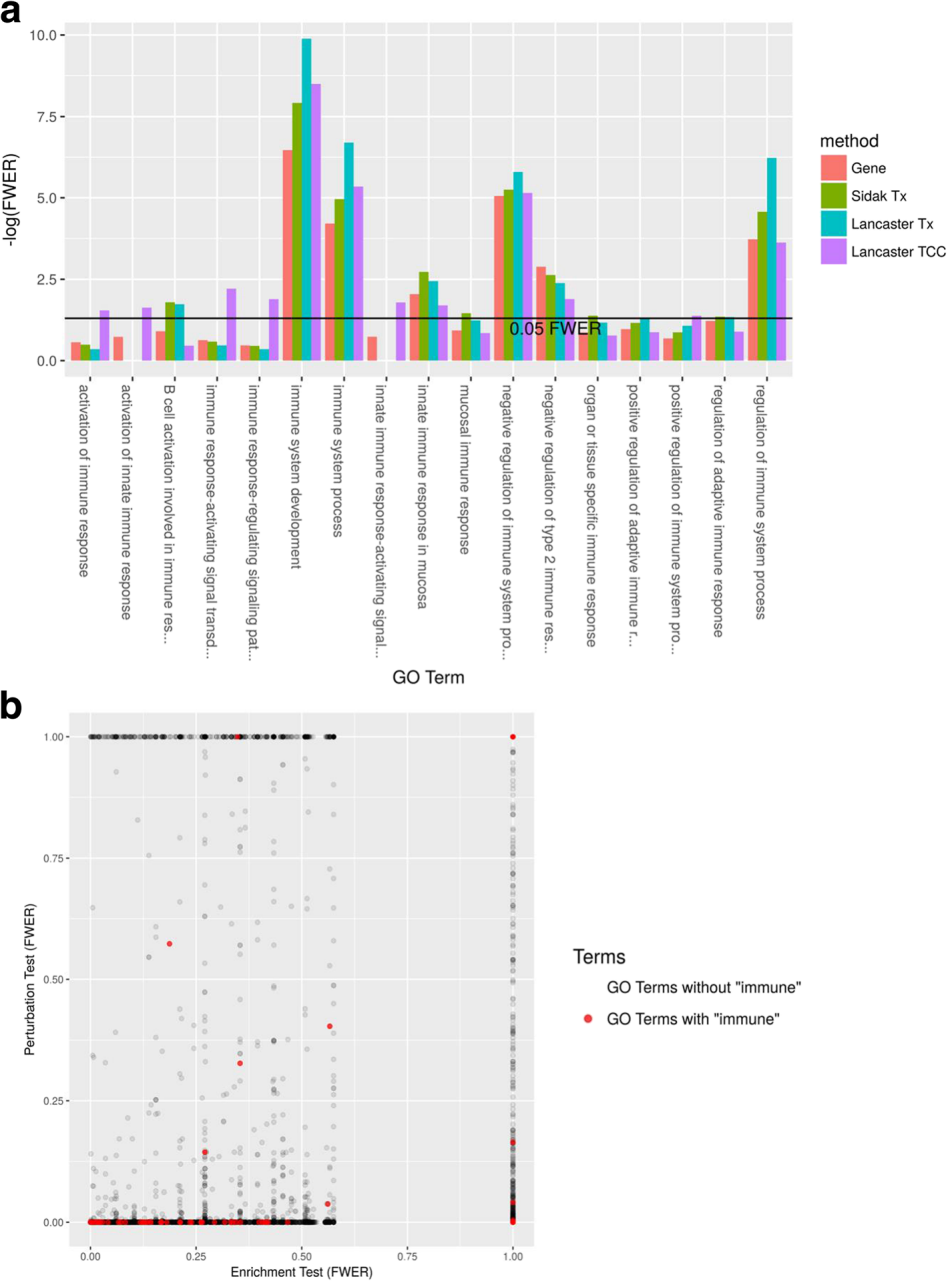
GO analysis based on *p* value aggregation. **a** Four aggregation methods (“Lancaster TCC,” “Lancaster Tx,” “Sidak Tx,” and “Gene”) were performed with sleuth to obtain gene-level differential expression analysis on response to dexamethasone treatment. The significant genes (FDR < 0.05) from each differential expression analysis were tested for GO enrichment (Fisher’s exact test) and Bonferroni-corrected. GO terms containing the word “immune,” for which at least one differential expression analysis provided a significant enrichment (FWER < 0.05), are shown with corresponding FWERs. Aggregation methods (“Lancaster TCC,” “Lancaster Tx,” and “Sidak Tx”) are better at detecting “immune” enrichment than *p* values derived from standard gene-level analysis (“Gene”). **b** TCC *p* values aggregated by GO term (“Perturbation Test”) reveal complementary information to classical GO enrichment (“Enrichment Test”)

We performed differential expression and GO analysis on recently published RNA-seq data that examined the effect of dexamethasone treatment on primary neural progenitor cells of embryonic mice ([25], GEO Series GSE95363). First, we performed differential expression using each of the four previously discussed aggregation methods to obtain differential gene lists (FDR < 0.05). (Additional file 1: Figure S4 compares differential expression with sleuth standard gene mode vs Lancaster aggregating TCC *p* values.) Then, we applied classical GO enrichment analysis to each method’s differential gene list. The Lancaster method applied to TCC-derived *p* values produced the differential gene list that is enriched for the most “immune”-containing GO terms (Fig. 5a). To apply the GO perturbation test, we performed further aggregation on the gene *p* values resulting from differential expression analysis to generate GO *p* values, resulting in a total of four GO perturbation tests. Each GO perturbation test resulted in a perturbed GO list (FWER < 0.05) that was more enriched for “immune”-containing GO terms than the corresponding enrichment test (FWER < 0.05) (Additional file 1: Figure S5).

To highlight some specific results, in the GO perturbation test based on aggregating TCC *p* values, we found 6396 GO terms (< 0.05 FWER) perturbed by dexamethasone treatment. Example terms at the top of the perturbed list included: system process (GO:0003008); response to stress (GO:0006950); metabolic process (GO:0008152); immune system process (GO:0002376); inflammatory response (GO:0006954); and response to hormone (GO:0009725). As a comparison, the corresponding classical enrichment analysis using Fisher’s exact test revealed 2123 enriched GO terms (< 0.05 FWER). Many of the perturbed GOs mentioned above were also enriched, but system process and inflammatory response were not (FWER = 0.27 and 1.00). In other words, an enriched ontology is likely perturbed, but not vice versa, and indeed, many “immune”-containing GO terms were perturbed but not enriched (Fig. 5b). These results suggest that perturbation analysis can be a useful and powerful complementary analysis to standard GO enrichment analysis.

## Discussion

We have shown that aggregating *p* values to obtain gene-level *p* values is a powerful and tractable method that provides biologically interpretable results. By using only the resulting *p* values from a differential expression analysis, aggregation bypasses issues of different variances and directions of change across constituent transcripts, allowing it to capture cancellation, domination, and collapsing events. All the examples of failure modes of traditional gene differential analysis showcased in Fig. 2 were successfully identified with the Lancaster method. Furthermore, performing the Lancaster method on TCC *p* values leverages the idea of pseudoalignment for RNA-seq, enabling a fast and model-free approach to differential analysis that circumvents numerous drawbacks of previous methods.

The method of *p* value aggregation is also extendable to testing other features of biological interest. We have demonstrated its utility for GO analysis to test for perturbation of gene ontologies, a complementary analysis that can be used in addition to existing GO enrichment tests. Aggregation can be performed hierarchically to maintain resolution at all levels including transcripts, genes, and GO terms. Further applications can include testing for intron retention, differential transcript start site (TSS) usage, and other use cases where aggregation of features is of interest. Finally, gene-level testing directly from TCC counts is particularly well-suited for single-cell RNA-seq analysis, where many technologies produce read distributions that are non-uniform across transcripts.

While this paper has focused on higher-order differential analysis, the complementary problem of differential analysis of individual transcripts can also benefit from some of the aggregation ideas described here. The stageR method, recently described in Van den Berge et al. [26], incorporates a two-step testing procedure in which an initial meta-analysis at the gene-level (using DEXSeq) is used to identify differential transcripts without losing power due to testing of all transcripts. The use of the Šidák method for aggregation of *p* values makes sense in that context, as it is desirable to identify genes with at least one differential isoform. However, it is possible that some of the methods we have introduced, including testing of TCCs and weighting, could be applied during the screening stage.

## Conclusions

Transcript differential analysis and gene differential analysis for RNA-seq have been two independent procedures up until now. Aggregating transcript *p* values with the Lancaster method to call gene differential expression not only outperforms other gene-level methods, it also retains information about transcript dynamics and produces one coherent analysis between transcripts and genes. This framework can be leveraged to study multiple resolutions of biology, such as performing a hierarchical analysis of transcripts, genes, and gene ontologies, or to bypass artifacts introduced at a particular resolution, such as obtaining gene-level results without transcript quantification by aggregating on TCCs.

## Methods

### Aggregation of *p* values

Fisher’s method aggregates *K p* values *p*_*1*_*,…, p*_*K,*_ which, under the null hypothesis, are independent and uniformly distributed between 0 and 1. Under the null hypothesis, the test statistic $T=\sum \limits_{i=1}^K-2\log \left({p}_i\right)$ is chi-squared distributed with degrees of freedom (*df) = 2K*. The aggregated *p* value is therefore $1-\phi \left(\sum \limits_{i=1}^K-2\log \left({p}_i\right)\right)$ where *ϕ* is the cumulative distribution function (CDF) of a chi-squared distribution with *df = 2 K* [27].

The Lancaster method [16] generalizes Fisher’s method for aggregating *p* values by introducing the possibility of weighting the *p* values with weights *w*_*1*_*,…,w*_*K*_. According to the Lancaster method, under the null hypothesis where all studies have zero effect, the test statistic $T=\sum \limits_{i=1}^K{\phi}_{wi}^{-1}\left({p}_i\right)$, where ${\phi}_{wi}^{-1}$ is the inverse CDF of the chi-squared distribution with *df = w*_*i*_, follows a chi-squared distribution with $df=\sum \limits_{i=1}^K{w}_i$. Fisher’s method is a specific instance of the Lancaster method where all *p* values are uniformly weighted by 2 and we found that the Lancaster method applied with a weighting scheme based on transcript counts outperformed Fisher’s method (Additional file 1: Figure S6).

We investigated whether the assumptions of Fisher’s and the Lancaster method, namely that *p* values are independent and uniformly distributed under the null hypothesis, apply to RNA-seq. Additional file 1: Figure S7 shows a distribution of the transcript *p* values for the dexamethasone RNA-seq data we examined. Aside from a peak close to 0, presumably corresponding to the differential transcripts, the *p* values appear to be uniformly distributed. Furthermore, the Additional file 1: Section 3 contains a walkthrough of the experiments we performed to test the independence between transcripts under the null hypothesis, showing that while transcripts of the same are not independent in general, the dependence is weak and does not lead to exaggerated *p* values or inflated FDRs (Additional file 1: Figures S8 and S9).

The Šidák method [28] utilizes a test based on the minimum *p* value *m = min(p*_*1*_*,…, p*_*K*_*)*, namely the adjustment *θ* = 1 – (1 − m)^*K*^. In the context of *K* isoforms with *p* values *p*_*1*_*,…, p*_*K*_, *θ* is the gene-level *p* value based on adjusting for the number of isoforms in the gene. If there are *M* genes, the adjustments will generate *p* values *θ*_*1, …,*_ *θ*_*M*_, which can be corrected for multiple testing. This method is similar to the perGeneQvalue result from DEXSeq [10], and while both methods control the FDR, the gene ranking is different between the two methods (Additional file 1: Section 2).

### Transcript differential analysis and aggregation

RNA-seq reads were quantified with kallisto v.0.43.1 to obtain transcript counts and abundances. These transcript counts were used as inputs in differential expression methods sleuth and DESeq2 in order to obtain transcript *p* values, which were then aggregated with the Lancaster method to obtain gene *p* values. sleuth and DESeq2 were run with their respective default filters and the Wald test. sleuth was run with 30 bootstraps. Transcripts filtered out from the differential expression analysis due to low counts were also filtered out from the *p* value aggregation. To obtain *p* value weights for the Lancaster method, we used as weights the mean expression level for the transcript extracted by the differential expression analysis (i.e. the mean_obs parameter in sleuth, the baseMean parameter in DESeq2). FDRs were calculated for the gene-specific *p* values using the Benjamini–Hochberg method. While we used the Wald test in this manuscript for obtaining transcript and gene differential expression analysis, we also tested the likelihood ratio test, which showed similar improvements with Lancaster aggregation and whose performance is comparable to the Wald test (Additional file 1: Figure S10).

### Transcript compatibility count differential analysis and aggregation

TCCs of RNA-seq reads were obtained with the kallisto *pseudo* option, which outputs a TCC matrix whose two dimensions are the number of samples and number of equivalence classes. Each TCC represents the RNA-seq counts corresponding to an equivalence class of transcripts. All TCCs corresponding to transcripts from more than one gene were filtered out from the analysis; 88% of reads were retained after applying this filter. The remaining TCCs were used to perform differential expression with sleuth [9] and DESeq2 [18] by using TCCs in lieu of transcript/gene counts. In order to use sleuth, we performed 30 bootstraps on TCCs, whose results were inputted into sleuth to estimate inferential variance. Non-expressed TCCs were filtered from the sleuth analyses and the default filter in DESeq2 was used. Both methods were performed with the likelihood ratio test because we found that the Wald test applied to TCCs reported overly liberal FDRs. The resulting TCC *p* values from the differential expression analysis were aggregated using the Lancaster method, with *p* value weights equal to the log-transformed mean counts normalized to 1. In other words, given *K* TCCs of the same gene with mean counts *t*_*1*_*, …, t*_*K*_, the weight for the *i*th TCC is ${w}_i=\frac{\log \left({t}_i+1\right)}{\sum_{j=1}^K\log \left({t}_j+1\right)\kern0.5em }$.

### Gene differential analysis

The aggregation methods were compared to standard gene-level differential analysis performed with sleuth and DESeq2. sleuth was run in gene mode with 30 bootstraps. DESeq2 was run on gene counts obtained using tximport [2] to aggregate transcript quantifications, except the case of 3’ QuantSeq dataset, where gene counts were obtained by summing reads that uniquely map to a gene. Both sleuth and DESeq2 were run with the Wald test and their respective default filters.

### Simulations

The simulations used to benchmark the method followed the approach of Pimentel et al. [9]. A null distribution consisting of the negative binomial model for transcript counts was learned from the Finnish female lymphoblastic cell lines subset of GEUVADIS [29]. A distribution of fold changes to the mean was learned from an experimental dataset from Trapnell et al. [6]; 20% of genes were chosen randomly to be differentially expressed, with fold changes of the transcripts assigned by rank-matching transcript abundances. Twenty simulations were performed, each with different randomly chosen sets of differentially expressed genes (for further details on the simulation structure, see [9]).

The simulations were quantified with kallisto v0.43.1 using an index constructed from Ensembl *Homo sapiens* GRCh38 cDNA release 79. Differential expression analyses were performed with sleuth and DESeq2 and then aggregated with various methods described above. Sensitivities and corresponding FDRs were calculated and then averaged across the 20 simulations. The average sensitivity at each average FDR was plotted with the mamabear package ([9], https://github.com/pimentel/mamabear).

### Rat alveolar epithelial cell stretching dataset analysis

We used a 3’ QuantSeq dataset (GEO Series GSE89024) of stretched and unstretched rat primary type I like alveolar epithelial cells. Five replicates for each condition were performed by the original experimenters, resulting in a total of ten single-end RNA-seq samples [21]*.* Reads were trimmed to remove poly-A tails with fqtrim-0.9.5 using the default parameters [30]. As discussed above in the “Methods” section under “Transcript compatibility count differential analysis and aggregation,” TCCs were obtained with the kallisto *pseudo* option, differential expression of TCCs was performed in sleuth, and TCC *p* values were aggregated with the Lancaster method. Because kallisto quantification is invalid for this non-uniform sequencing dataset and it cannot be used to provide bootstrap estimates of inferential variance required for sleuth, we used DESeq2’s default pipeline to perform gene differential analysis, summing all reads mapping uniquely to a gene to obtain gene counts.

### Dexamethasone dataset analysis

We analyzed a dataset (GEO Series GSE95363) consisting of reads derived from RNA-seq on primary mouse neural progenitor cells extracted from two regions of the brain, from female and male embryonic mice, and with and without dexamethasone treatment. Three replicates were performed for each of the eight combinatorial conditions, resulting in a total of 24 single-end RNA-seq samples [25]. As detailed above in “Transcript differential analysis and aggregation,” samples were quantified with kallisto v0.43.1 (default kmer length 31, with 30 bootstraps per sample), using an index constructed from Ensembl *Mus musculus* GRCm38 cDNA release 88. Within sleuth, a linear model with three parameters (gender, brain region, and treatment) was constructed, a Wald test was performed to test for effect of treatment on transcript expression, and the resulting *p* values were aggregated. As detailed above in “Transcript compatibility count differential analysis and aggregation,” TCCs were obtained with kallisto v0.43.1 using the *pseudo* option, differential expression of TCCs was performed in sleuth, and the resulting *p* values aggregated. On this dataset, we also performed the sleuth’s standard gene pipeline (detailed in “Gene differential analysis”) and the Sidak aggregation method, resulting in a total of four different aggregation methods.

Each method’s significant gene list, thresholded at FDR < 0.05, was inputted into a classical GO analysis to test for GO enrichment. topGO_2.26.0 [31] was invoked to perform Fisher’s exact test, using gene ontologies drawn from GO.db_3.4.0 and mouse gene annotations drawn from org.Mm.eg.db_3.4.0 [32]. Furthermore, the gene *p* values from each aggregation method were used in a GO perturbation test. In the GO perturbation test, gene *p* values are weighted by the counts mapping uniquely to the gene and aggregated with the Lancaster method, using the ontology-to-gene mappings provided by topGO. The GO *p* values were Bonferroni corrected to obtain FWER.

### Software versions

DESeq2 1.14.1 and sleuth 0.29.0 were used in R version 3.4.1 to perform differential analyses. Tximport 1.2.0 was used to sum transcript counts within genes to perform gene-level differential expression with DESeq2. We implemented Fisher’s method and Lancaster method with the chisq and gamma functions in the R Stats Package. A lightweight R package containing the functionality for performing *p* value aggregation with Fisher’s, Lancaster and Šidák methods, which is applicable generally to outside the domain of RNA-Seq, is available on CRAN as “aggregation” (https://cran.r-project.org/web/packages/aggregation/). Our method to perform gene-level differential analysis via Lancaster aggregation of transcript *p* values has been implemented in sleuth. Scripts to reproduce the figures and results of the paper are available at http://github.com/pachterlab/aggregationDE/.

## Additional file

Additional file 1: Supplementary material. (PDF 13757 kb)
