# Supplementary material for: Gene-level differential analysis at transcript-level resolution
Source: Genome Biol. 2018 Apr 12;19:53. doi: 10.1186/s13059-018-1419-z (PMC5896116; doi:10.1186/s13059-018-1419-z)
Supplement: Supplementary file 1 — Supplementary material. (PDF 13757 kb) [file 13059_2018_1419_MOESM1_ESM.pdf]

Supplementary materials for "Gene-level differential analysis at transcript-level resolution"

Lynn Yi Harld Pimentel Nicolas L. Bray* Lior Pachter*

January 16, 2018

1 Converting transcript counts to gene abundances

Consider a gene with two transcripts t_1 and t_2 of lengths l_1 and l_2 and a two-condition experiment where X_1^i reads originate from transcript t_1 in experiment i ($i \in \{1, 2\}$ and X_2^i ($i \in \{1, 2\}$ reads originate from transcript t_2. The current recommended approach to gene-level differential analysis is to compute a gene abundance for each condition as follows:

$$Y^i \propto \left(\frac{l_1 + l_2}{2}\right) \cdot \left(\frac{X_1^i}{l_1} + \frac{X_2^i}{l_2}\right). \tag{1}$$

The sum $\frac{X_1^i}{l_1} + \frac{X_2^i}{l_2}$ computes gene abundance as the sum of transcript abundance, and the factor $\frac{l_1+l_2}{2}$ produces a "gene count" to be used in differential expression methods such as DESeq2 (Love *et al.*, 2014) that perform shrinkage. The formula may contain extra constants related to sequencing depth. Note that

$$\frac{Y^1}{Y^2} \propto \frac{X_1^1 l_2 + X_2^1 l_1}{X_1^2 l_2 + X_2^2 l_1}. \tag{2}$$

This estimate of log-fold change avoids the problems discussed in (Trapnell *et al.*, 2013). However in terms of variance estimation, the "gene count" $Y^i \propto X_1^i l_2 + X_2^i l_1$ can be problematic when $l_1 \neq l_2$. Specifically if (without loss of generality) $l_1 < l_2$ then variance in X_1^i may be amplified by l_2, an undersirable property since there is no reason why the variance contribution of one transcript should depend on the length of another.

2 Šidák aggregation

Let T be the set of transcripts and G the set of genes. For each $g \in G$, denote the number of transcripts in g by $|g| = |\{t : t \in g\}|$. Let P_t be the uniform random variable denoting the p-value output by a transcript-level differential analysis method under the null hypothesis of no differential abundance of transcript t. For a specific experiment, let p_{tg} denote the p-value obtained from testing for differential abundance of transcript t in gene g by that method. Let $m_g = min_{t \in g} p_{tg}$ and set $u_g = 1 - (1 - m_g)^{|g|}$.

For each $g \in G$, let H_g be the null hypothesis that no transcript $t \in g$ is differentially abundant. Assume that the hypotheses H_g are independent, and additionally that for each g the hypotheses resulting in the values p_{tg} are independent (i.e. the random variables $\{P_t\}_{t \in g}$ are independent).

Claim 1. *The Benjamini-Hochberg step-up procedure applied to the values $\{u_g\}_{g \in G}$ controls the FDR for the $|G|$ null hypotheses $\{H_g\}_{g \in G}$.*

Proof: Denote by M_g the p-value that under the null hypothesis H_g the smallest among the $\{P_t\}_{t \in g}$ is less than or equal to m_g. Note that

$$M_g = \mathbb{P}\left(\bigcup_{t \in g} (P_t \leq m_g)\right) = 1 - \prod_{t \in g} \mathbb{P}\left(P_t > m_g\right) = 1 - (1 - m_g)^{|g|}. \tag{3}$$

Therefore under the null hypotheses the values u_g are uniformly distributed. Given a significance threshold α, the Benjamini-Hochberg step-up procedure identifies the largest k such that if the u_g are ordered from smallest to largest as $u_1 \leq u_2 \leq u_3 \leq \cdots$ then

$$u_k \leq \frac{k}{|G|}\alpha. \tag{4}$$

The procedure then rejects the null hypotheses corresponding to $u_1, \ldots, u_k$. The assumption that the hypotheses H_g are independent guarantees that Benjamini-Hochberg procedure has a false discovery rate bounded by α.

Note that for small values of m_g, $1 - (1 - m_g)^{|g|} \approx |g| m_g$ by Taylor approximation. The use of $|g| m_g$ instead of u_g would be equivalent to a Bonferroni correction at the gene-level prior to the Benjamini-Hochberg procedure. As described, the u_g can be viewed as a Šidák correction (Šidák, 1967).

In the DEXSeq program (Anders *et al.*, 2012) there is a similar approach that is implemented for aggregation, but that is slightly different. Instead of applying the Šidák correction to the minimm p-value from each gene and sorting genes according to the u_g values, in DEXSeq genes are sorted directly according to the m_g values. While the DEXSeq approach to controlling the false discovery rate is correct (Reyes *et al.*, 2012), the ordering according to m_g is not equivalent to the ordering according to u_g and has a drawback. Specifically, genes with many isoforms are more likely, by chance, to produce a small p-value in one of their isoforms, and this has two consequences. First, the most highly ranked genes (i.e. smallest value of m_g) will tend to have more isoforms, and second, while DEXSeq controls the FDR there are more likely to be false positives with small m_g values rather than small u_g values, so that the "FDR budget" is consumed more quickly.

When simulating p-values according to a null distribution (i.e. each transcript in the genome receives a p-value that is uniformly distributed) for the *Mus musculus* transcriptome (GRCm38 release 88), we found that the average number of isoforms among the top 100 ranked genes according to m_g is 6.89 (median = 5) versus 2.77 for u_g (median = 1). For context, the average number of transcripts per gene in the *Mus musculus* transcriptome is 3.1 with a median of 1.

3 Applicability of the Lancaster method to RNA-Seq

Transcripts of the same gene are not in general independent from each other since they may be biological co-regulated and are quantified from the same pool of reads. Therefore, we performed a series of experiments demonstrating that the extent of transcript dependence is limited and that the assumption of independence in performing the Lancaster method is a reasonable approximation. Furthermore we show that the application of the Lancaster method to aggregating transcript p-values does not inflate the false discovery rate (FDR) or false positive rate (FPR).

3.1 Independence of transcript p-values

We performed an *in silico* experiment to measure distribution of p-values under the null hypothesis. To construct an instance of the null hypothesis, we chose six samples randomly from within the GEUVADIS set of samples performed on Finnish women and performed a 3v3 differential expression analysis using sleuth on transcripts. A pair of transcript p-values was randomly selected from each gene and scatter-plotted (Supplementary Figure 8), showing that transcript p-values appear linearly uncorrelated (Pearson correlation of 0.064), and other than a point of high density of low-low p-value pairs, appear uniform and independent by first-order visualization.

To test directly for independence, a chi-square test was performed on a contingency table constructed on these p-value pairs by binning p-values uniformly in 0.1 intervals. The chi-square test resulted in a p-value of 7.5e$-$8, supporting the idea that p-values of isoforms within a gene are not independent, which is expected. However, when p-values within the $(0, 0.1)$ interval are excluded from the chi-square test, the chi-square test for independence resulted in a p-value of 0.74, consistent with what we noticed upon visualization and demonstrating that transcript p-value dependence is largely limited to small p-value regimes but is otherwise independent.

3.2 Control of false positive rates and false discovery rates

One reasonable critique of applying the Lancaster method to RNA-Seq is that even weak dependence between transcripts under the null hypothesis leads to exaggerated gene p-values and an inflated false positive rate (FPR) and false discovery rate (FDR), especially when small transcript p-values are aggregated. To address this critique, we performed an experiment to test the effect of transcript dependence on the FPR and FDR under the null hypothesis. In each trial of our experiment, we randomly chose six samples from the same batch within the Finnish women GEUVADIS samples and compared the numbers of false positives (p-value < 0.05) and false discoveries (q-value < 0.05) found in gene mode compared the numbers found with the Lancaster method using sleuth. We performed 20 such trials and tabulated the results in Supplementary Figure 9. In all 20 trials, the number of false positives were reduced with Lancaster aggregation (paired t-test, p-value = 6.5e$-$6), and in 14 trials, the number of false discoveries were reduced (paired t-test, p-value = 0.141). The null simulation results demonstrate that the Lancaster method resulted in a reduction

of the FPR and FDR, not an inflation, and are consistent with the findings in the perturbation simulation results (Figure 3, Supplementary Figures 1-3) that the Lancaster method reported more accurate and conservative FDRs than other methods.

4 Independent and Correlated Effect Simulations

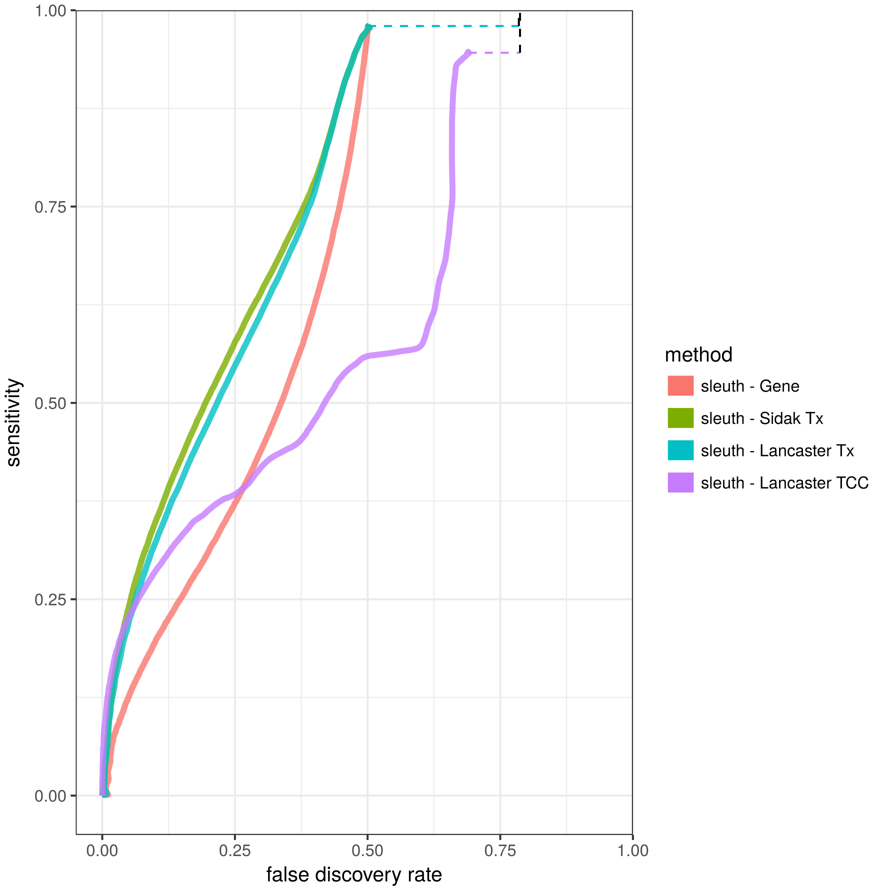

Supplementary Figure 1a: Independent effect simulation.

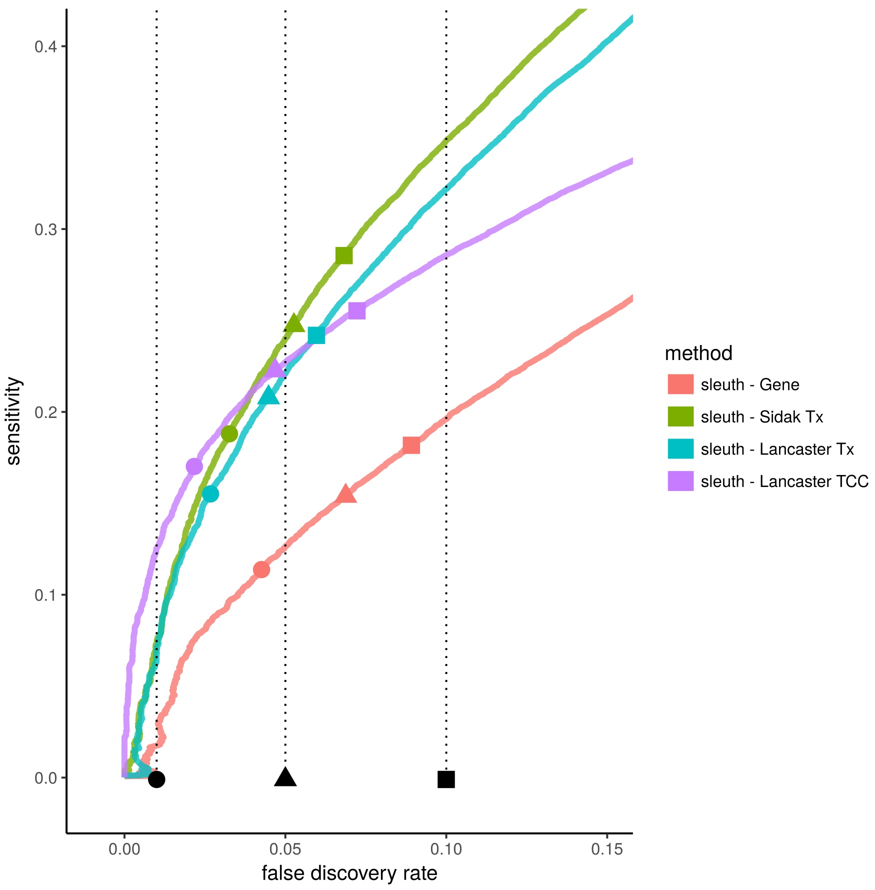

Supplementary Figure 1b: Independent effect simulation (zoomed in).

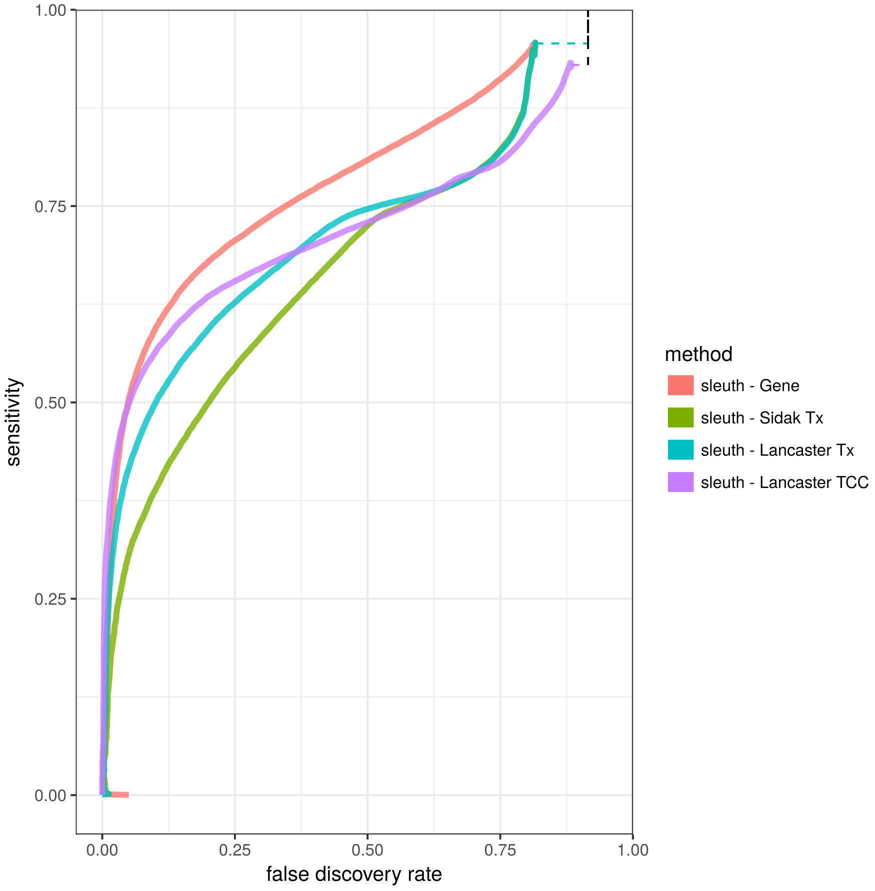

Supplementary Figure 2a: Correlated effect simulation.

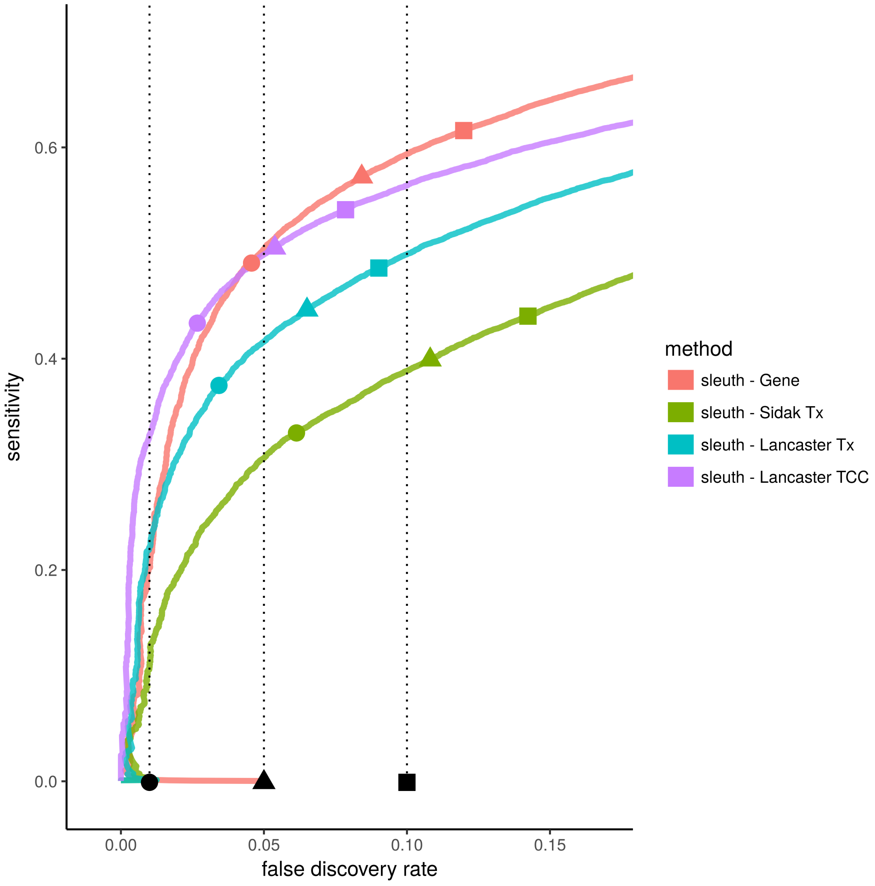

Supplementary Figure 2b: Correlated effect simulation (zoomed in).

5 DESeq2 Performance on Simulations

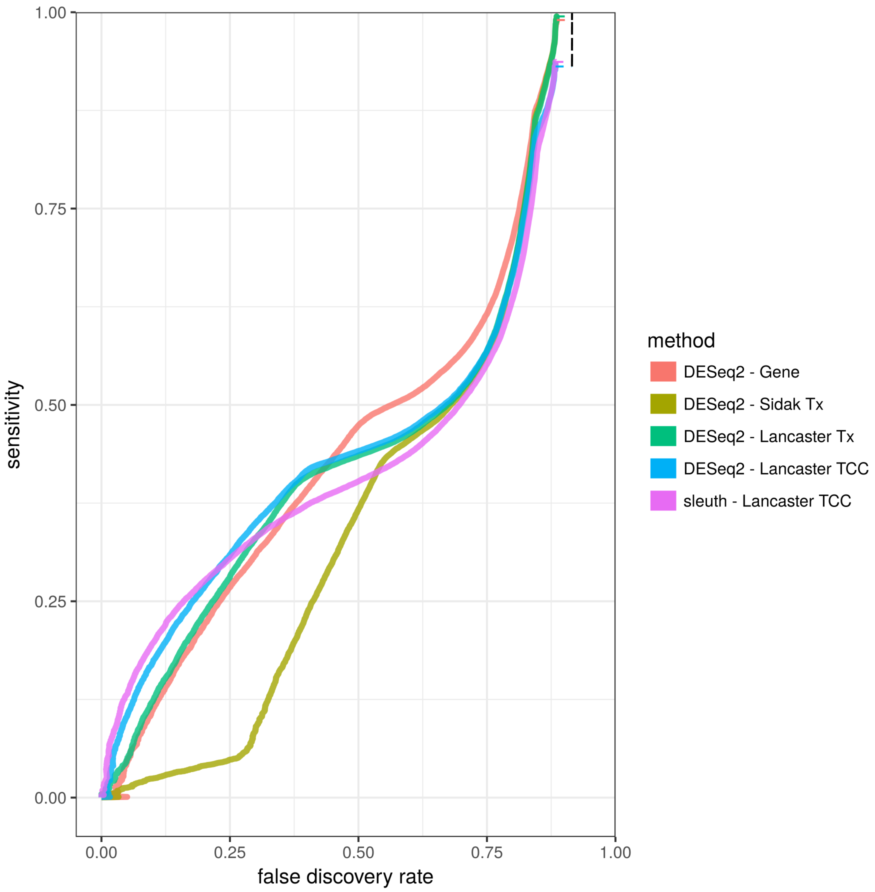

Supplementary Figure 3a: Experimental effect simulation with DESeq2.

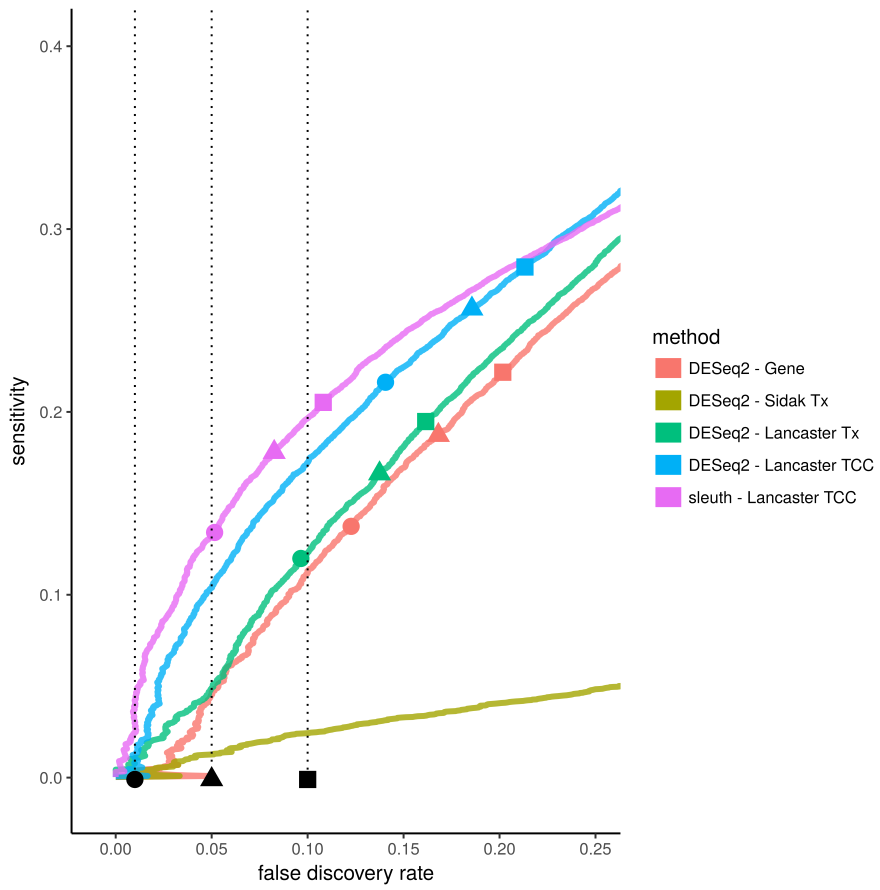

Supplementary Figure 3b: Experimental effect simulation with DESeq2 (zoomed in).

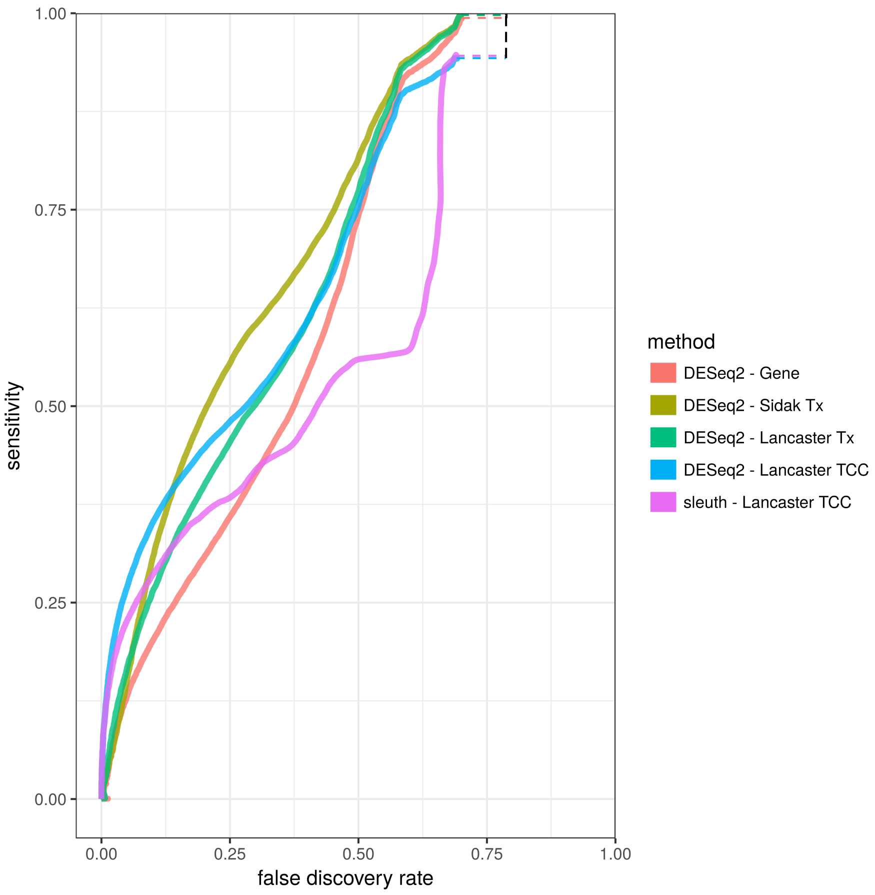

Supplementary Figure 3c: Independent effect simulation with DESeq2

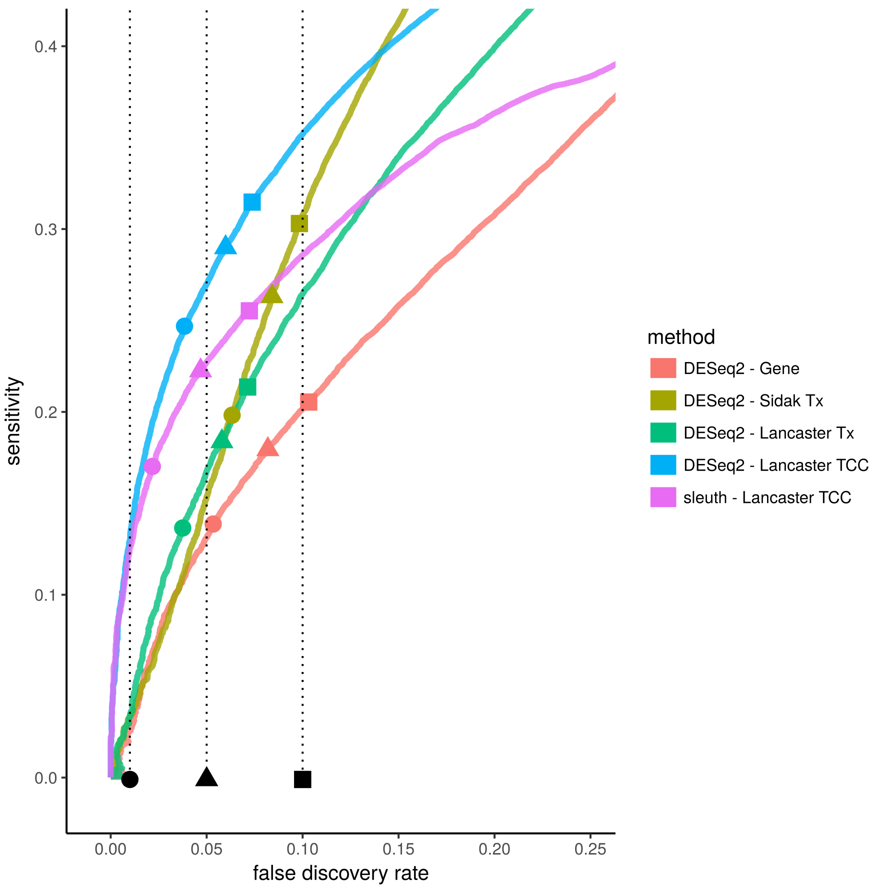

Supplementary Figure 3d: Independent effect simulation with DESeq2 (zoomed-in).

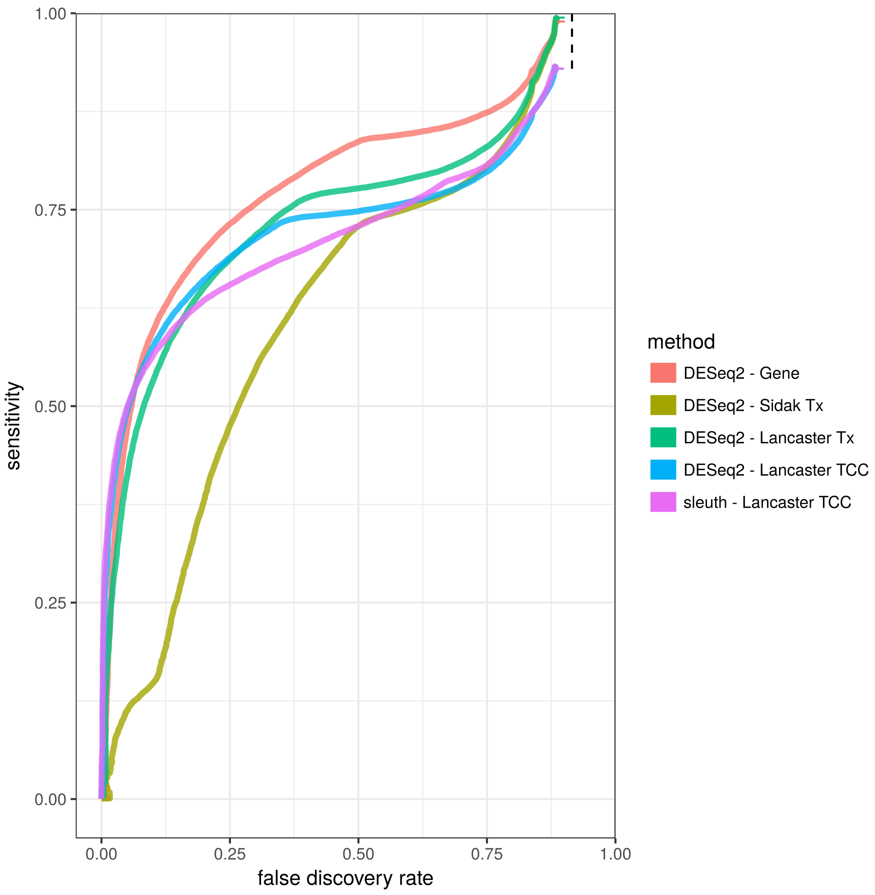

Supplementary Figure 3e: Correlated effect simulation with DESeq2.

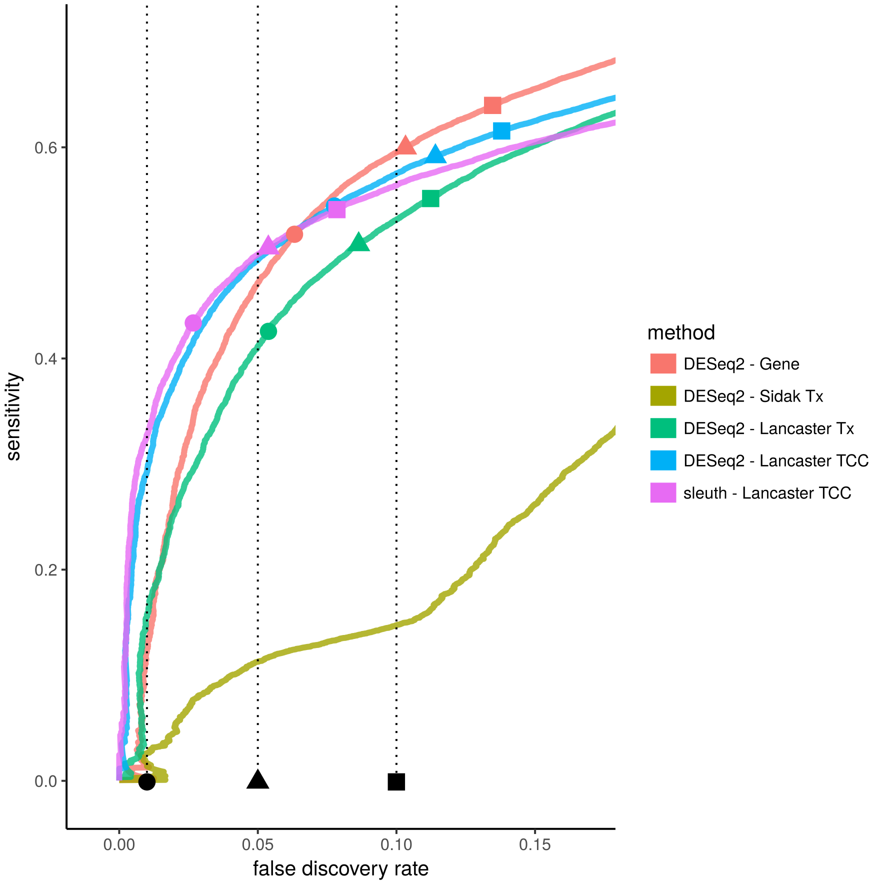

Supplementary Figure 3f: Correlated effect simulation with DESeq2 (zoomed-in).

6 Additional Supplementary Figures

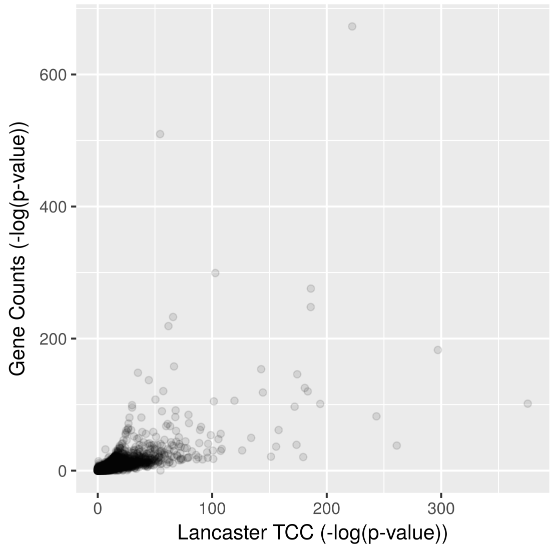

Supplementary Figure 4: Log-log scatterplot of the p-values from a standard gene-level analysis in sleuth and the p-values from Lancaster aggregating sleuth-derived TCC p-values. The dexamethasone dataset was used, with p-values corresponding to the significance that the gene was differentially expressed in dexamethasone vs. vehicle treatment. 2772 differential genes (FDR $<$ 0.05) were discovered by standard gene analysis compared to the 4336 significant genes (FDR $<$ 0.05) discovered by Lancaster aggregation on TCCs.

Enrichment of 'Immune' GO Terms in GO Analysis Results

	GO Enrichment Test	GO Perturbation Test
Gene	0.99	2.94E-05
Sidak - Tx	0.94	0.00049
Lancaster - Tx	0.99	0.0005
Lancaster - TCC	0.96	0.0896

Supplementary Figure 5: Enrichment of 'Immune' GO Terms in GO Analysis Results. Four differential expression analyses ('Gene', 'Sidak Tx', 'Lancaster - Tx', and 'Lancaster TCC') were performed on the dexamethasone dataset using sleuth. A classical GO enrichment test and a GO perturbation test were performed on results from each of the four methods, resulting in eight GO analyses. Fisher's exact test was performed to test for GO enrichment in the significant gene list (p-val < 0.05) identified by each differential expression method. To perform the GO perturbation test, the p-values of genes in the GO term were weighted by gene counts and aggregated with the Lancaster method. GO p-values were Bonferroni corrected and a significant GO list (FWER $<$ 0.05) was constructed for each method. Each GO list was then tested for enrichment in 'immune'-containing GO terms with Fisher's exact test and the resulting p-values were tabulated.

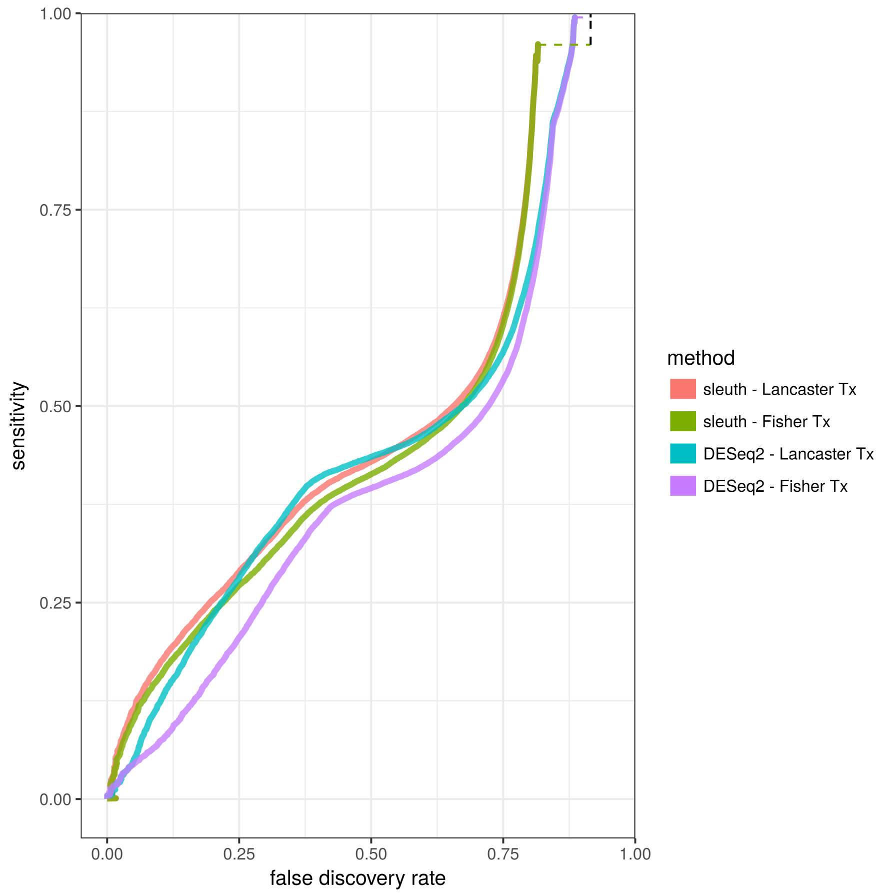

Supplementary Figure 6a: Fisher's method and the Lancaster method were compared based on their performance on the experimental effect simulation. Transcript p-values derived from sleuth and DESeq2 were aggregated with Fisher's method and the Lancaster method. The results from 20 experiments were averaged.

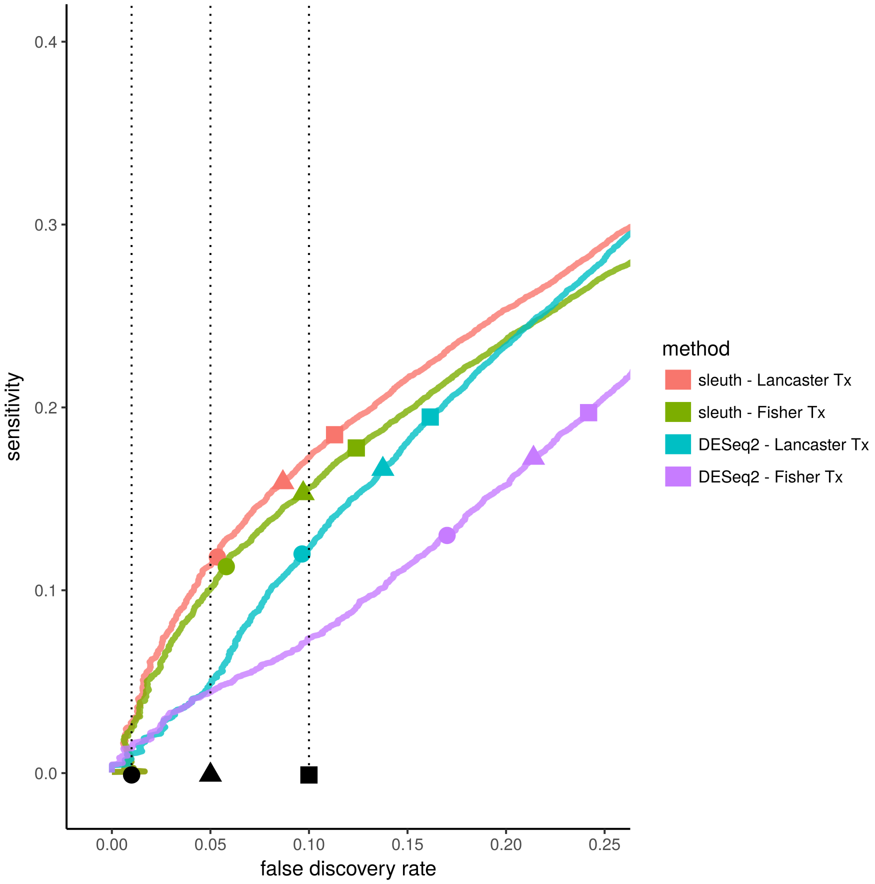

Supplementary Figure 6b: Fisher's method vs. the Lancaster method (zoomed-in)

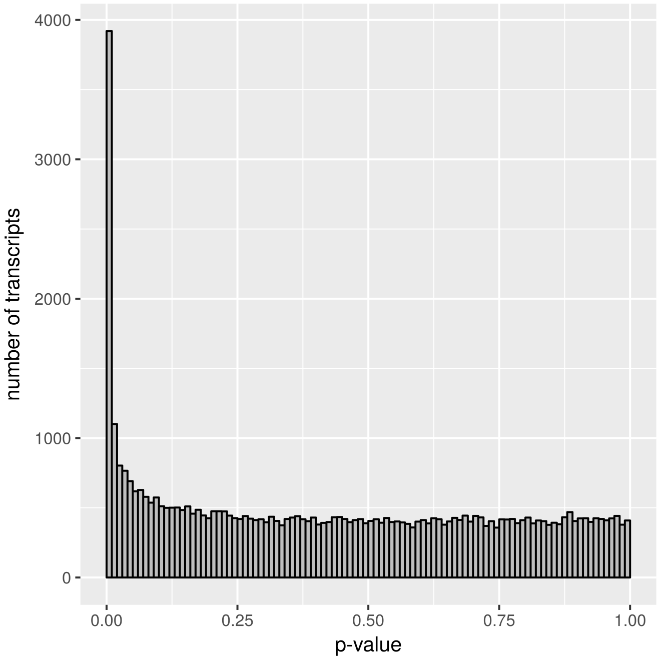

Supplementary Figure 7: Distribution of transcript p-values from the dexamethasone data set.

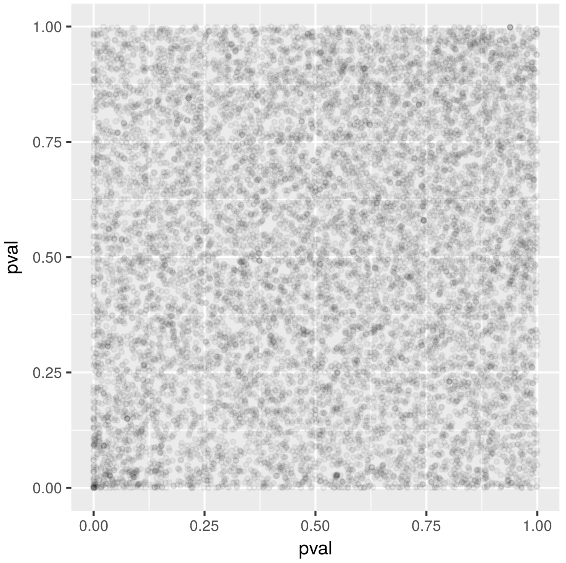

Supplementary Figure 8: Distribution of transcript p-values pairs from the same gene under the null hypothesis.

Trial #	False Positives (p-value < 0.05) Gene mode	Lancaster aggregation	Difference	False Discoveries (q-value < 0.05) Gene mode	Lancaster aggregation	Difference
1	1463	428	-1035	15	16	1
2	1778	553	-1225	25	19	-6
3	8858	4024	-4834	1966	2010	44
4	8360	3353	-5007	1798	1495	-303
5	2567	742	-1825	83	81	-2
6	5123	1952	-3171	245	285	40
7	1866	623	-1243	56	41	-15
8	1421	447	-974	14	20	6
9	1541	386	-1155	8	12	4
10	1380	393	-987	24	22	-2
11	3904	1325	-2579	236	203	-33
12	1154	381	-773	38	27	-11
13	2096	484	-1612	45	24	-21
14	1626	418	-1208	22	16	-6
15	1269	386	-883	45	33	-12
16	1175	340	-835	55	34	-21
17	1355	383	-972	24	23	-1
18	909	221	-688	27	19	-8
19	706	174	-532	6	8	2
20	2738	792	-1946	57	56	-1
**Mean**	2564.5	890.3	**-1674.2**	239.5	222.2	**-17.3**
**p-value (paired t-test)**			**6.546E-06**			**0.141**

Supplementary Figure 9: Comparisons of the number of false positives and discoveries obtained by sleuth gene mode versus Lancaster aggregation on sleuth-derived transcript p-values. Each of the twenty null hypothesis trials is simulated by performing 3 vs. 3 differential expression analyses in sleuth of batch-corrected samples from Finnish women in the GEUVADIS dataset.

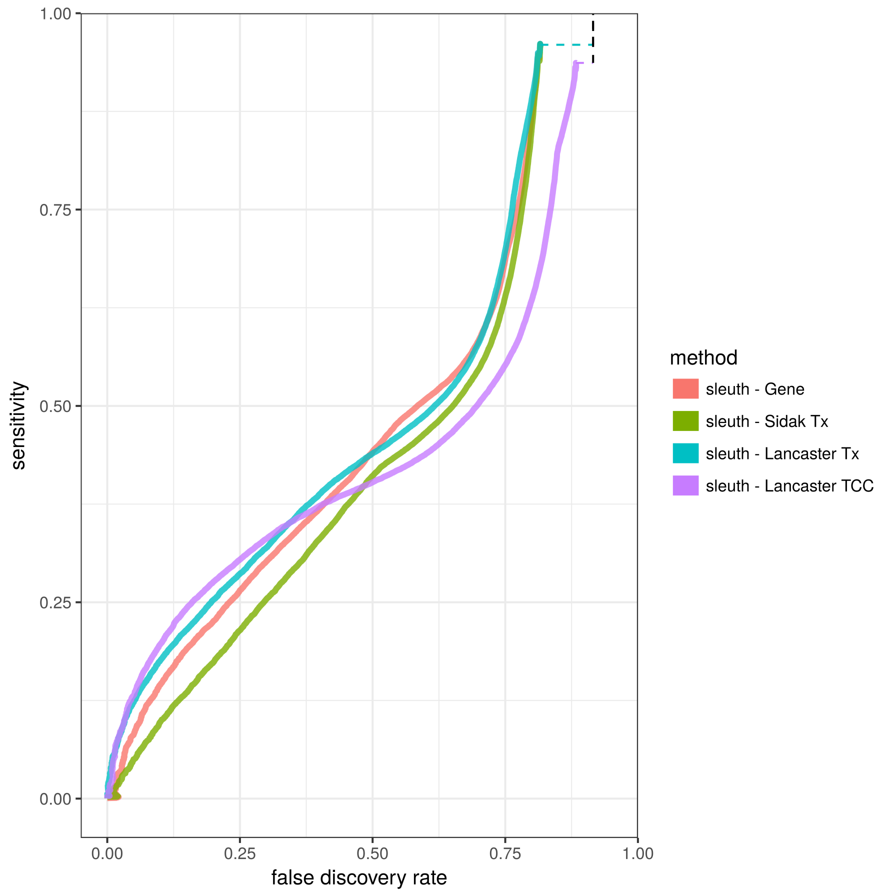

Supplementary Figure 10a. Performance of the likelihood ratio test on the experimental effect simulation. Instead of the Wald test, sleuth was invoked using the likelihood ratio test in gene mode ('sleuth - Gene') and on transcripts. Transcript p-values from the likelihood ratio test were aggregated with the Sidak correction ('sleuth - Sidak') or the Lancaster method ('sleuth - Lancaster Tx'). For sake of comparison, 'sleuth - Lancaster TCC,' which is always performed with the likelihood ratio test, is displayed.

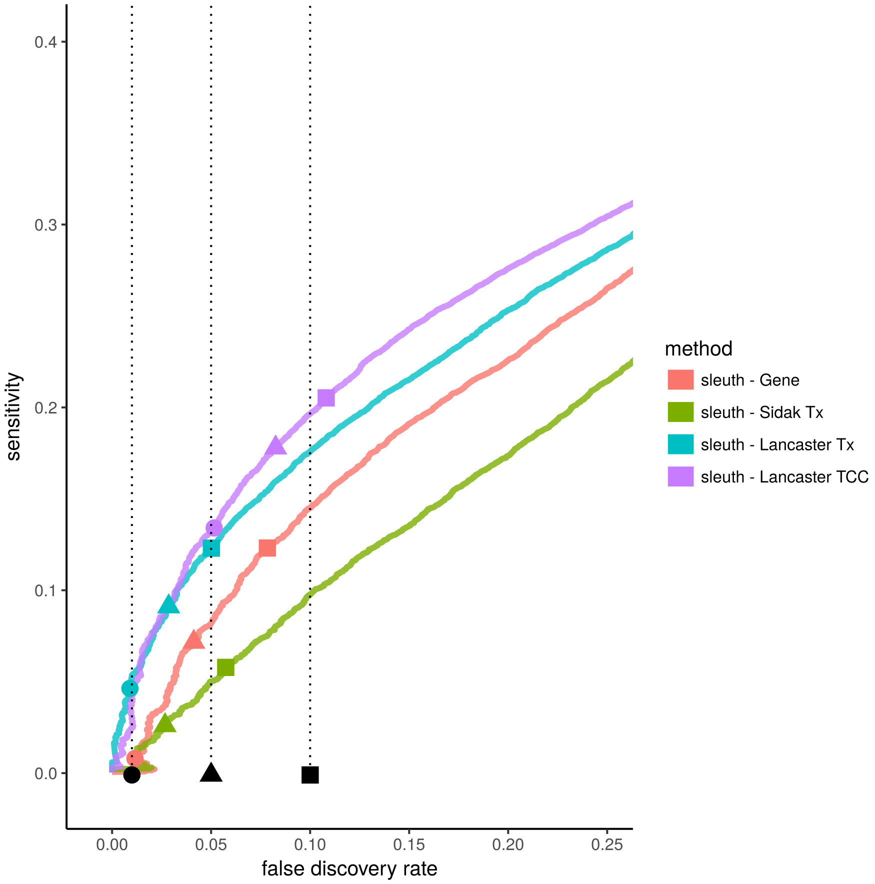

Supplementary Figure 10b. Performance of the likelihood ratio test on the experimental effect simulation (zoomed-in).

7 References

Love MI, Huber W, Anders S. Moderated estimation of fold change and dispersion for RNA-seq data with DESeq2. Genome Biology. 2014, 15, pp. 550. doi:10.1186/s13059-014-0550-8.

Trapnell C, Hendrickson DG, Sauvageau M, Goff L, Rinn JL, Pachter L. Differential analysis of gene regulation at transcript resolution with RNA-seq. Nat Biotechnol. 2013 Jan;31(1):46-53. doi: 10.1038/nbt.2450.

Šidák, ZK. Rectangular Confidence Regions for the Means of Multivariate Normal Distributions. Journal of the American Statistical Association. 1967, 62, p 626–633. doi: 10.1080/01621459.1967.10482935

Anders S, Reyes A, Huber W. Detecting differential usage of exons from RNA-seq data. Genome Res. 2012 Oct;22(10):2008-17. doi: 10.1101/gr.133744.111.

Reyes A, Anders S, Huber W. Inferring differential exon usage in RNA-Seq data with the DEXSeq package. Updated 2016 Nov. https://bioconductor.org/packages/release/bioc/vignettes/DEXSeq/inst/doc/DEXSeq.pdf.
